# Effects of osteosynthesis of the bony thorax in the context of polytrauma compared to conservative treatment: a systematic review

**DOI:** 10.1007/s00068-024-02760-z

**Published:** 2025-01-24

**Authors:** Karolina Dahms, Jan Volmerig, Julia Dormann, Eva Steinfeld, Kelly Ansems, Heidrun Janka, Maria-Inti Metzendorf, Carina Benstoem

**Affiliations:** 1 Department of Intensive Care Medicine and Intermediate Care Medical Faculty, RWTH Aachen University, Pauwelsstr. 30, D-52074 Aachen, Germany; 2 Institute of General Practice, Medical Faculty of the Heinrich-Heine- University Dusseldorf, Dusseldorf, Germany; 3 Clinic for Thoracic Surgery, Evang. Kliniken Essen-Mitte, University of Duisburg-Essen, Essen, Germany

**Keywords:** Rib fixation, Intensive care unit, ICU, Polytrauma, Multiple trauma, SSRF

## Abstract

**Purpose:**

Osteosynthesis seems to have effects regarding clinical outcomes in trauma patients. However, current knowledge on chest wall osteosynthesis in polytrauma patients is insufficient, leaving its potential unanswered. Therefore, the objective of this systematic review is to assess the safety and effects of chest wall osteosynthesis compared to conservative treatment on clinical outcomes in adult polytrauma patients.

**Methods:**

We searched PubMed to identify completed and ongoing studies from inception of each database to May, 2022. We included systematic reviews including RCTs comparing chest wall osteosynthesis to conservative treatment in adult polytrauma patients.

**Results:**

We included one RCT with 50 patients (n_osteosyntheses_ = 25, n_control_ = 25, median age 37.4 years, 82% male). We found that surgical rib fixation makes little or no difference to in-hospital mortality compared to conservative treatment (RR 2.00, 95% CI 0.40 to 9.95; RD 80 more per 1,000, 95% CI 48 fewer to 716 more; 1 study, 50 participants, low quality of evidence). We found that surgical rib fixation makes little or no difference to the need for mechanical ventilation compared to conservative treatment (RR 0.90, 95% CI -0.66 to 1.23; RD 80 fewer per 1,000, 95% CI 272 fewer to 184 more; 1 study, 50 participants, low certainty of evidence).

**Conclusion:**

There is limited evidence regarding chest wall osteosynthesis compared to conservative treatment in polytrauma patients. One RCT shows no effect of surgical rib fixation compared to conservative treatment regarding mortality and clinical status, but a potential benefit regarding ICU length of stay.

## Introduction

Currently, the literature investigating the treatment of injuries of the bony thorax, with or without involvement of the sternum or clavicle, possibly with consecutive instability, is heterogeneous [1–5]. Despite partly concordant tendencies in favor of osteosynthetic treatments of the chest wall, there are still very divergent conclusions regarding surgical indication, timing, and technique of performance as well as investigated parameters (e.g., duration of ventilation). Notably, the majority of studies focus on specific populations or isolated injuries, with a paucity of robust evidence specifically addressing polytrauma patients. Furthermore, most available studies were conducted several years ago, underscoring a gap in updated evidence that reflects current clinical practices or advancements in surgical techniques.

Studies on osteosynthetic treatment of (more or less) “isolated” rib series fractures as well as unstable thoraces have shown advantages of osteosynthesis in ventilation time, development of pneumonia or chest deformity, treatment time in the intensive care unit, and hospitalization time [3, 5–6].

Polytrauma patients, however, represent a distinct and highly complex patient population that should be considered separately from those with isolated thoracic injuries. These patients often present with a constellation of life-threatening injuries and unique physiological challenges, which may alter the efficacy and applicability of treatment approaches derived from other populations [7]. Despite this, there is little or no evidence available regarding the treatment of the bony thorax in this critical patient group. One retrospective study from 2015, including polytrauma patients (ISS ≥ 16) with unstable thorax found no significant difference between osteosynthesis and conservative treatment with respect to duration of ventilation, tracheostomy, pneumonia, and acute respiratory distress syndrome (ARDS) rate, whereas ICU and hospital length of stay increased significantly, even with secondary exclusion of patients with craniocerebral trauma [4]. Importantly, this limited evidence base not only lacks breadth but also fails to capture more recent advancements in the understanding and management of polytrauma.

This reflects the lack of sufficient evidence specific to this patient population, which currently precludes clear conclusions regarding the superiority of osteosynthesis versus conservative treatment strategies. Current recommendations remain consensus-based or extrapolated from studies in other patient groups, particularly those with isolated rib series fractures [8, 9].

By addressing this evidence gap, this review aims to provide a foundation for more focused and evidence-driven clinical recommendations for this distinct patient population. Therefore, the objective of this systematic review is to assess the safety and effects of chest wall osteosynthesis compared to conservative treatment on clinical outcomes in adult polytrauma patients.

## Methods

This review is part of the guideline project ‘S3-Leitlinie Intensivmedizin nach Polytrauma’ (AWMF Nr. 040 − 014) guided by the German Interdisciplinary Association of Critical Care and Emergency Medicine (Deutsche Interdisziplinäre Vereinigung für Intensiv- und Notfallmedizin, DIVI) and the German Society for Anaesthesiology and Intensive Care Medicine (Deutsche Gesellschaft für Anästhesiologie und Intensivmedizin, DGAI). The aim was to summarize the current evidence in the field of polytrauma to formulate specific recommendations. All studies that were carried out as part of this project used the same methodology which was consented within the guideline group.

### Eligibility criteria

We included studies comparing chest wall osteosynthesis to conservative treatment in adult polytrauma patients admitted to the ICU that met the following inclusion criteria:

- age of the included patients is ≥ 18 years.
- polytrauma is present and is defined as: a simultaneous injury to multiple body regions or organ systems, at least one or more of which, in combination, is life-threatening [10].
- the study type is a or systematic review that includes RCTs.
- the language of publication is English or German.
- it is not a multiple publication without additional information.
- the publication can be obtained as a full text.
- comparison of chest wall osteosynthesis with conservative treatment.

## Search strategy

We conducted a systematic search in PubMed via the Cochrane Library in May 2022. Records were included from inception of each database to May 18, 2022 with no restrictions on the language of publication. Details of our search strategy are provided in the Appendix No 1. In addition, we searched reference lists of included studies to identify other potentially eligible studies.

## Study selection

We imported the records from the systematic search into the Rayyan Systematic Review App (Rayyan, Cambridge, MA, USA). Three authors independently screened the titles and abstracts of all potential studies. Included full-text study publications were retrieved, imported into Excel and screened by two authors independently. Reasons for exclusion of ineligible studies were recorded. Any disagreements were resolved through discussion or, if required, consultation with a third author.

## Data collection process

One reviewer extracted study and outcome data into a customized data collection form developed in Microsoft Excel, which was checked by a second investigator. Any disagreements were resolved by discussion or by consulting a third review author if necessary.

The following data were obtained:

1. Study characteristics: authors, publication date, and study design.
2. Participants characteristics: number of included participants, gender, age.
3. Intervention: intervention, control.
4. Clinical outcomes: all-cause mortality (day 28, day 60, time to-event, and up to longest follow-up), clinical status (duration of mechanical ventilation, need for mechanical ventilation), length of stay, serious adverse events (SAE), adverse events (AE), infections, quality of life.

We transmitted the outcome data into a statistical software (RevMan 5.3, Cochrane, London, England). Missing data resulted in the exclusion of the study in the analyses of the missing outcome.

## Study risk of bias assessment

The risk of bias of included studies was assessed by two authors independently using the Risk of Bias 2 (RoB 2) tool (Cochrane, London, England). This tool addresses five domains of bias (randomization process, deviations from intended interventions, missing outcome data, measurement of the outcome, selection of the reported results). The signalling questions of the tool were used to make a judgement according to the available options. We used the algorithms proposed in RoB 2 to assign each domain and the overall risk of bias to a level of bias: low risk of bias, some concerns, high risk of bias. Any disagreements between reviewers were resolved by discussion or by involvement of another author.

### Synthesis methods

To summarize demographics, we used descriptive statistics. A meta-analyses was performed only, if the clinical and methodological characteristics of individual studies were sufficiently homogeneous. For all analyses, we used a statistical software (RevMan 5.3, Cochrane, London, England). Data entry into the Rev-Man software was checked by a second review author for accuracy. Outcome data were pooled using the random-effects model, as we anticipated that true effects would be related, but not the same for the studies included in our review. For dichotomous data, we performed analyses using the Mantel–Haenszel method under a random-effects model to report pooled risk ratios (RR) with 95% confidence intervals (CI). For continuous outcomes, we calculated mean differences with 95% CIs. Forest plots were provided to summarize the effects from individual studies.

## Certainty assessment

We used GRADEpro Guideline Development Tool Software (McMaster University and Evidence Prime Inc., Hamilton, Ontario, Canada) to create a summary of findings table and evaluated the certainty of the evidence using the GRADE approach for interventions evaluated in RCTs.

## Results

### Study selection

**Fig. 1:**
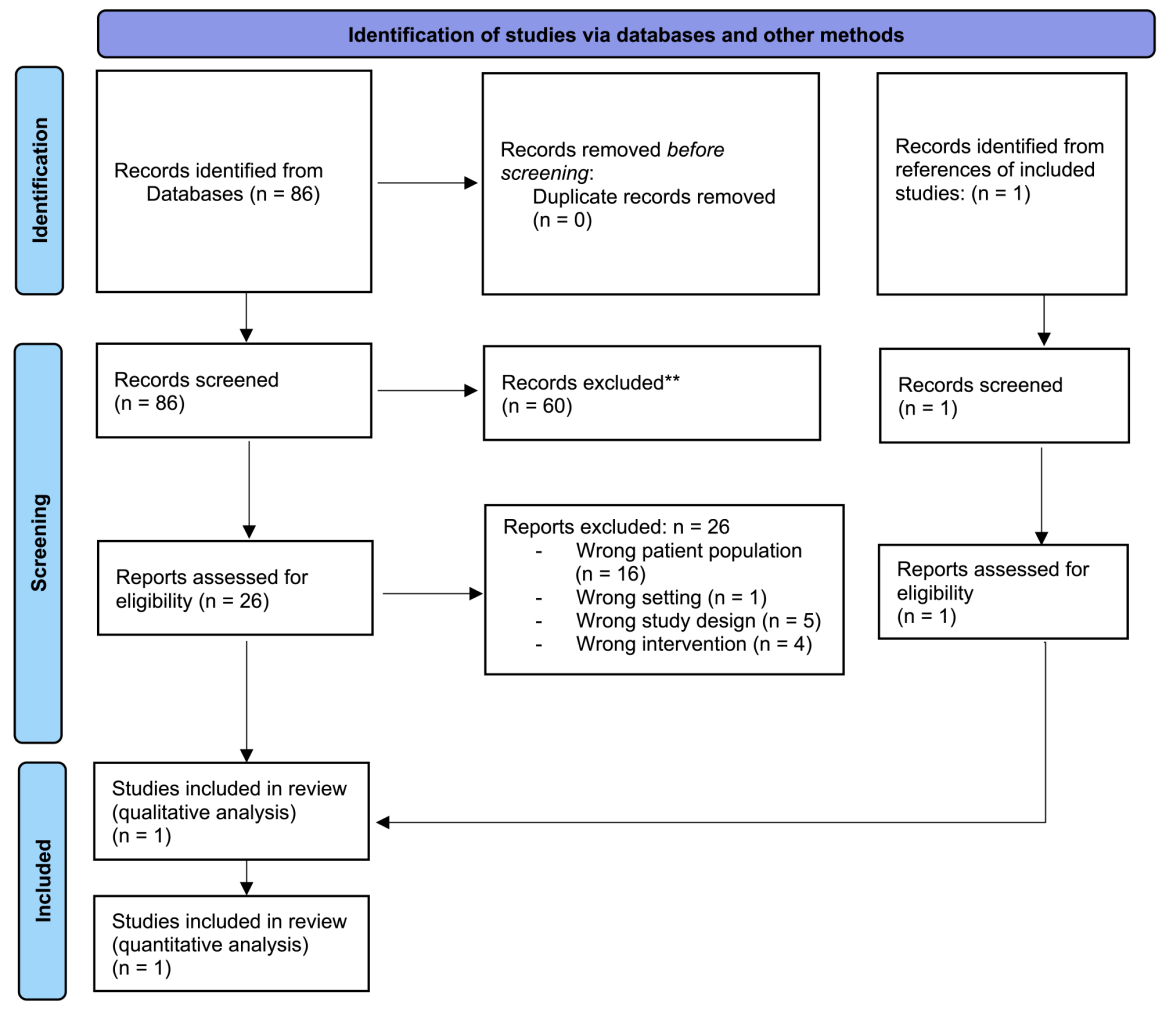
Flowchart of the systematic review selection process

The search identified 86 records, which were screened based on title and abstract. Sixty studies did not meet the prespecified inclusion criteria and were excluded. We screened the full texts and trial register entries of the remaining 26 references. All records were excluded for different reasons (Fig. 1). One study was detected via references of screened record and included for quantitative synthesis [11].

### Study characteristics

One RCT [10] with a total of 50 participants (median age 40.5 years, 82% male) with polytrauma met the criteria for inclusion. The RCT used a parallel-group design and compared surgical and nonsurgical management of polytrauma patients with an unstable chest wall (‘flail chest’). Study characteristics are presented in table ​1.

**Table 1:** Study characteristics

Study (Year)	Study design	No of patients	Median age (Range) in years	Gender (m/f)	Intervention	Control
Liu et al. (2019)	RCT	*N* = 50 Operative: *N* = 25 Non-operative: *N* = 25	Operative: 42 (25–58) Non-operative: 39 (24–56)	Operative: 21/4 20/5	Operative fixation with U-plate	Conservative treatment

### Risk of bias in studies

The overall risk of bias of the RCT was some concerns for all outcomes due to a lack of a pre-specified analysis plan, no concealment or blinding.

### Results of individual studies

Surgical rib fixation compared to conservative treatment in adult patients with polytrauma (summary of findings Table 2).

**Table 2:** Summary of findings table

Outcomes	№ of participants (studies) Follow-up	Certainty of the evidence (GRADE)	Relative effect (95% CI)	Anticipated absolute effects	
				Risk with non-surgery	Risk difference with surgery
In-hospital mortality	50 (1 RCT)	⨁⨁◯◯ Low^a, b^	RR 2.00 (0.40 to 9.95)	80 per 1.000	80 more per 1.000 (48 fewer to 716 more)
Need for mechanical ventilation	50 (1 RCT)	⨁⨁◯◯ Low^a, b^	RR 0.90 (0.66 to 1.23)	800 per 1.000	80 fewer per 1.000 (272 fewer to 184 more)
ICU Length of stay	(1 RCT)	⨁⨁◯◯ Low^a, c^	ICU Length of stay was significantly shorter in the intervention group with surgery than in the group without surgery (*p* = 0.032). Surgical group, days in median (IQR): 10 (7–12) Non-surgical group, days in median (IQR): 12 (9–15)		
Hospital length of stay	(1 RCT)	⨁⨁◯◯ Low^a, c^	There was no significant difference in the length of hospital stay between the intervention and the control group (*p* = 0.44). Surgical group, days in median (IQR): 21 (17–25) Non-surgical group, days in median (IQR): 22 (17–26)		

*The risk in the intervention group (and its 95% confidence interval) is based on the assumed risk in the comparison group and the relative effect of the intervention (and its 95% CI).

CI: confidence interval; RR: risk ratio

a. risk of bias due to no concealment of assignment; no blinding of patients and staff

b. imprecision due to very wide confidence intervals, few participants and only one study

c. imprecision due to few participants and only one study

### Mortality

**Fig. 2:**
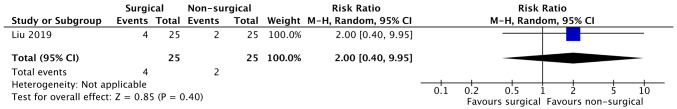
Forest plot describing the difference between surgical rib fixation compared to conservative treatment regarding all-cause mortality

One study reported in-hospital mortality for 50 participants (Fig. 2). We found that surgical rib fixation makes little or no difference to in-hospital mortality compared to conservative treatment. (Risk ratio (RR) 2.00, 95% confidence interval (CI) 0.40 to 9.95; risk difference (RD) 80 more per 1,000, 95% CI 48 fewer to 716 more; 1 study, 50 participants, low certainty of evidence). The reason for downgrading was imprecision due to few patients, only one study and concerns regarding risk of bias due to no concealment and no blinding.

### Clinical status: need for mechanical ventilation

**Fig. 3:**
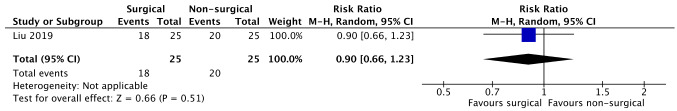
Forest plot describing the difference between surgical rib fixation compared to conservative treatment regarding Need for mechanical ventilation

One study reported duration of mechanical ventilation for 50 participants (Fig. 3). We found that surgical rib fixation makes little or no difference to the need for mechanical ventilation compared to conservative treatment (RR 0.90, 95% CI -0.66 to 1.23; RD 80 fewer per 1,000, 95% CI 272 fewer to 184 more; 1 study, 50 participants, low certainty of evidence). The reason for downgrading was imprecision due to few patients, only one study and concerns regarding risk of bias due to no concealment and no blinding.

### Length of stay

One study reported ICU length of stay for 50 participants (Table 3). We found that surgical rib fixation had a significant effect compared to no surgical intervention (10 days [IQR 7–12 vs. 12 days [IQR 9–15], *p* = 0.032; low certainty of evidence). The reason for downgrading was imprecision due to few patients, only one study, very wide confidence intervals and concerns regarding risk of bias due to no concealment and no blinding.

**Table 3:** Length of stay in polytrauma patients

Median number of days mean (IQR) Surgical group	No of patients Surgical group	Median number of days mean (IQR) Nonsurgical group	No of patients Nonsurgical group	*P*- value
**ICU Length of stay**				
10 (7–12)	25	12 (9–15)	25	0,032
**Hospital length of stay**				
21 (17–25)	25	22 (17–26)	25	0,44

One study reported hospital length of stay for 50 participants (Table 3). We found that surgical rib fixation makes little or no difference to hospital length of stay compared to no surgical intervention (21 days [IQR 17–25] vs. 22 days [IQR 17–26], *p* = 0.44; low certainty of evidence). The reason for downgrading was imprecision due to few patients, only one study, very wide confidence intervals and concerns regarding risk of bias due to no concealment and no blinding.

We found no data regarding serious adverse events, adverse events, and quality of life.

## Discussion

In this review, one RCT comparing surgical rib fixation with conservative treatment in 50 adult polytrauma patients was included. The study findings revealed that there were no significant differences in in-hospital mortality, the requirement for mechanical ventilation, and the length of hospital stay when comparing surgical rib fixation with conservative treatment. However, when assessing the length of stay in the ICU, surgical rib fixation appeared to have a favorable impact, leading to a reduction in ICU duration compared to conservative treatment.

A 2016 systematic review, which does not focus on polytrauma patients, similarly concluded that mortality rates did not differ between the intervention groups (operative vs. non-operative management of flail chest) [1]. This review as well as the review performed by Cataneo et al. also revealed a positive effect of surgical rib fracture fixation and surgical intervention for flail chest on pneumonia rate, duration of mechanical ventilation, duration of ICU, hospital stay and tracheostomy rate [1, 5]. In contrast, a retrospective analysis of the German Trauma Registry database, which examined polytrauma patients from 2008 to 2017 using a matched pairs analysis involving 395 pairs, suggested a reduction in mortality, but an extended duration of mechanical ventilation, ICU stay, and overall hospital stay after surgical stabilization [12]. These seemingly contradictory findings may be attributed to differences in patient populations, the extent and severity of associated injuries, and variations in surgical timing and techniques.

With regard to critically ill trauma patients, a prospective, controlled evaluation of surgical stabilization of rib fractures (SSRF) showed improved outcomes [13]. Particularly with regards to respiratory failure, tracheostomy, and duration of ventilation, the group who underwent surgery demonstrated superior outcomes when compared to the non-operative group. Furthermore, two retrospective cohort studies focused on osteosynthetic treatment in polytrauma patients. These studies revealed that patients who underwent surgery experienced a prolonged need for ventilation and extended hospital or intensive care stays with different advantages observed in individual treatment parameters (e.g. overall survival, incidence of pneumonia). Notably, factors such as the timing of surgical treatment and the extent of pulmonary contusions were considered prognostic parameters [4, 14]. This underscores the importance of individualized treatment strategies and highlights how nuanced factors can substantially influence outcomes.Additionally, no high-quality recent studies focus specifically on polytrauma patients, highlighting a critical gap in the literature. This patient population is particularly complex, as polytrauma often involves severe injuries to multiple organ systems, dynamic physiological derangements, and complications such as pulmonary contusions, hemorrhagic shock, and acute respiratory distress syndrome (ARDS). These factors make the management of bony thorax injuries in polytrauma patients uniquely challenging and necessitate evidence tailored to their specific needs. Current findings derived from isolated thoracic injury cohorts cannot simply be extrapolated to polytrauma patients, given their fundamentally different clinical trajectories and outcomes. In summary, numerous studies show no or limited comparability due to differences in reported parameters and divergent inclusion and exclusion criteria.

It is important to recognize the limitations of this systematic review. The search period of this systematic review was 2 years ago, necessitating a search update. Further, we focused only on systematic reviews including RCTs. However, the limited availability of RCTs results in a restricted relevance of these findings into today’s clinical practice. The low level of certainty into the evidence, stemming from some concerns regarding the risk of bias within the included study and imprecision of the results, further diminishes the robustness of the conclusions. Given these considerations, this systematic review highlights the urgent need for additional high-quality RCTs specifically addressing polytrauma patients. Evidence is urgently required not only to clarify the comparative effectiveness of surgical versus conservative treatment but also to guide critical clinical decisions in a population where missteps can have profound implications for survival and quality of life. Understanding the specific needs and challenges associated with polytrauma patients is essential to optimize their management and improve outcomes in this highly vulnerable population.

## Conclusion

There is limited evidence regarding chest wall osteosynthesis compared to conservative treatment in polytrauma patients. One RCT shows no effect of surgical rib fixation compared to conservative treatment regarding mortality and clinical status, but a potential benefit regarding ICU length of stay.

## Appendix no 1. Search strategy

**Total hits: 86**.

**- nach Deduplication: 86**.

1. **PubMed**.

#1.

“Thoracic Injuries“[mh] OR “Flail Chest“[mh] OR “Rib Fractures“[mh] OR (“Multiple Trauma“[mh] AND “Thorax“[mh]) OR “thoracic injur*“[tw] OR “thoracic trauma“[tw] OR “chest trauma“[tw] OR “chest wall trauma“[tw] OR “thorax trauma“[tw] OR “chest injur*“[tw] OR “chest wall injur*“[tw] OR “flail chest*“[tw] OR “stove-in chest*“[tw] OR “rib fracture*“[tw] OR “costal fracture*“[tw] OR “sternal fracture*“[tw] OR “sternal injur*“[tw] OR “sternum fracture*“[tw] OR “sternum injur*“[tw] OR (“multiple fracture*“[tiab] AND “rib“[tiab])

#2.

“Fracture Fixation“[mh] OR “fixation“[tw] OR osteosynthe*[tw] OR “stabilization“[tw] OR “stabilisation“[tw] OR surgic*[tiab] OR surger*[tiab] OR operati*[tiab].

#3.

#1 AND #2 (1’670) (8891).

#4.

systematic[sb].

#5.

#3 AND #4.

(86)

**2. CENTRAL (via CRSO)**.

#1 (xxxxxxxxxxxxxxxxx): TI, AB, KY.

#2 (polytrauma* OR multiple trauma* OR major trauma* OR multiple injur*):TI, AB, KY.

#3 #1 AND #2.

*= x*.

3.

**Web of Science (Science Citation Index und Emerging Citation Index)**.

1. TI=(xxxxx) OR AB=(xxxxxxxxxxxx).
2. TI=(polytrauma* OR multiple trauma* OR major trauma* OR multiple injur*) OR AB=(polytrauma* OR multiple trauma* OR major trauma* OR multiple injur*).
3. 1 AND 2.
4. TI=(random* OR placebo OR trial OR groups) OR AB=(random* OR placebo OR trial OR groups).
5. TI=(meta analysis OR systematic review) OR AB=(search* OR meta analysis OR medline OR systematic review).
6. 4 OR 5.
7. 3 AND 6.

*= x*.

## Data Availability

No datasets were generated or analysed during the current study.

## Abbreviations

ARDS: acute respiratory distress syndrome
CI: confidence interval
DGAI: German Society for Anaesthesiology and Intensive Care Medicine (Deutsche Gesellschaft für Anästhesiologie und Intensivmedizin)
DIVI: German Interdisciplinary Association of Critical Care and Emergency Medicine (Deutsche Interdisziplinäre Vereinigung für Intensiv- und Notfallmedizin)
ICU: intensive care unit
ISS: injury severity score
MD: mean difference
MH: Mantel–Haenszel
RCT: randomized controlled trial
RD: risk difference
RoB: risk of bias
RR: risk ratio

## References

[1] Schuurmans J, Goslings JC, Schepers T. Operative management versus non-operative management of rib fractures in flail chest injuries: a systematic review. Eur J Trauma Emerg Surg. 2017;43:163–8. 27572897 10.1007/s00068-016-0721-2 PMC5378742

[2] Kocher GJ, Sharafi S, Azenha LF, Schmid RA. Chest wall stabilization in ventilator-dependent traumatic flail chest patients: who benefits? Eur J Cardiothorac Surg. 2017;51(4):696–701. 28007867 10.1093/ejcts/ezw365

[3] Swart E, Laratta J, Slobogean G, Mehta S. Operative treatment of Rib fractures in Flail chest injuries: a Meta-analysis and cost-effectiveness analysis. J Orthop Trauma. 2017;31(2):64–70. 27984449 10.1097/BOT.0000000000000750

[4] DeFreest L, Tafen M, Bhakta A, Ata A, Martone S, Glotzer O, Krautsak K, Rosati C, Stain SC, Bonville D. Open reduction and internal fixation of rib fractures in polytrauma patients with flail chest. Am J Surg. 2016;21(4):761–7. 10.1016/j.amjsurg.2015.11.014 26899958

[5] Cataneo AJ, Cataneo DC, de Oliveira FH, Arruda KA, El Dib R, de Oliveira Carvalho PE. Surgical versus nonsurgical interventions for flail chest. Cochrane Database Syst Rev. 2015;(7):CD009919. 10.1002/14651858.CD009919.pub2 PMC9189492 26222250

[6] Kasotakis G, Hasenboehler EA, Streib EW, Patel N, Patel MB, Alarcon L, Bosarge PL, Love J, Haut ER, Como JJ. Operative fixation of rib fractures after blunt trauma: a practice management guideline from the Eastern Assoiation for the surgery of Trauma. J Trauma Acute Care Surg. 2017;82(3):618–26. 28030502 10.1097/TA.0000000000001350

[7] Iyengar KP, Venkatesan AS, Jain VK, Shashidhara MK, Elbana H, Botchu R. Risks in the management of Polytrauma patients: clinical insights. Orthop Res Rev. 2023;15:27–38. 36974036 10.2147/ORR.S340532 PMC10039633

[8] Kong LW, Huang G-B, Yi YF, Du DY. The Chinese consensus for surgical treatment of traumatic rib fractures 2021 (C-STTRF 2021). Chin J Traumatol. 2021;24(6):311–9. 34503907 10.1016/j.cjtee.2021.07.012 PMC8606596

[9] Pieracci FM, Majercik S, Ali-Osman F, Ang D, Doben A, Edwards JG, French B, Gasparri M, Marasco S, Minshall C, Sarani B, Tisol W, VanBoerum DH, White TW. Consensus statement: Surgical stabilization of rib fractures rib fracture colloquium clinical practice guidelines. Injury. 2017;48(2):307–21. 27912931 10.1016/j.injury.2016.11.026

[10] Tscherne H. The treatment of the severly injured at an Emergency Station. Chirurg. 1966;37:249–52. 5995039

[11] Liu T, Liu P, Chen J, Xie J, Yang F, Liao Y. A Randomized Controlled Trial of Surgical Rib fixation in Polytrauma patients with Flail. Chest J Surg Res. 2019;242:223–30. 31100568 10.1016/j.jss.2019.04.005

[12] Schulz-Drost S, Krinner S, Langenbach A, Oppel P, Lefering R, Taylor D, Hennig FF, Mauerer A. Concomitant Sternal fracture in Flail Chest: an analysis of 21,741 Polytrauma patients from the TraumaRegister DGU^®^. Thorac Cardiovasc Surg. 2017;65(7):551–9. 28187475 10.1055/s-0037-1598194

[13] Pieracci FM, Lin Y, Rodil M, Synder M, Herbert B, Tran DK, Stoval RT, Johnson JL, Biffl WL, Barnett CC, Cothren-Burlew C, Fox C, Jurkovich GJ, Moore EE. A prospective, controlled clinical evaluation of surgical stabilization of severe rib fractures. J Trauma Acute Care Surg. 2016;80(2):187–94. 26595710 10.1097/TA.0000000000000925

[14] Becker L, Schulz-Drost S, Spering C, Franke A, Dudda M, Lefering R, Matthes G, Bieler D. Committee on Emergency Medicine, Intensive Care, Trauma Management (Sektion NIS) of the German Trauma Society (DGU). Effect of surgical stabilization of rib fractures in polytrauma: an analysis of the TraumaRegister DGU(^®^). Eur J Trauma Emerg Surg. 2020;48(4):2773–81. 10.1007/s00068-021-01864-0 PMC9360126 35118558

